# Endocytic Trafficking of Nanoparticles Delivered by Cell-penetrating Peptides Comprised of Nona-arginine and a Penetration Accelerating Sequence

**DOI:** 10.1371/journal.pone.0067100

**Published:** 2013-06-26

**Authors:** Betty R. Liu, Shih-Yen Lo, Chia-Chin Liu, Chia-Lin Chyan, Yue-Wern Huang, Robert S. Aronstam, Han-Jung Lee

**Affiliations:** 1 Department of Natural Resources and Environmental Studies, National Dong Hwa University, Hualien, Taiwan; 2 Department of Laboratory Medicine and Biotechnology, Tzu Chi University, Hualien, Taiwan; 3 Department of Life Sciences, Tzu Chi University, Hualien, Taiwan; 4 Department of Chemistry, National Dong Hwa University, Hualien, Taiwan; 5 Department of Biological Sciences, Missouri University of Science and Technology, Rolla, Missouri, United States of America; University of Pécs Medical School, Hungary

## Abstract

Cell-penetrating peptides (CPPs) can traverse cellular membranes and deliver biologically active molecules into cells. In this study, we demonstrate that CPPs comprised of nona-arginine (R9) and a penetration accelerating peptide sequence (Pas) that facilitates escape from endocytic lysosomes, denoted as PR9, greatly enhance the delivery of noncovalently associated quantum dots (QDs) into human A549 cells. Mechanistic studies, intracellular trafficking analysis and a functional gene assay reveal that endocytosis is the main route for intracellular delivery of PR9/QD complexes. Endocytic trafficking of PR9/QD complexes was monitored using both confocal and transmission electron microscopy (TEM). Zeta-potential and size analyses indicate the importance of electrostatic forces in the interaction of PR9/QD complexes with plasma membranes. Circular dichroism (CD) spectroscopy reveals that the secondary structural elements of PR9 have similar conformations in aqueous buffer at pH 7 and 5. This study of nontoxic PR9 provides a basis for the design of optimized cargo delivery that allows escape from endocytic vesicles.

## Introduction

The translocation of the transactivator of transcription (Tat) protein of the human immunodeficiency virus type 1 (HIV-1) into cells depends on a sequence that contains eleven amino acids (amino acid sequence: YGRKKRRQRRR) [1]. Using this basic amino acid-rich sequence as a guide, many small peptides were designed that possess a similar membrane penetrating potential [2]. These peptides, dubbed cell-penetrating peptides (CPPs), may be amphipathic, hydrophobic or cationic [3]. CPPs can facilitate the delivery of cargoes, including DNAs, RNAs, proteins and nanoparticles, into living cells [2], [4], [5]. More than 843 varieties of CPPs have been catalogued on a CPP site (http://crdd.osdd.net/raghava/cppsite/) [6].

Although CPPs have recently gained much attention as powerful tools to introduce exogenous molecules into cells, their cellular uptake pathways and subsequent intracellular trafficking are still not fully understood. Studies have suggested that CPPs utilize multiple pathways for cellular entry [7]–[9]. Endocytosis and direct membrane translocation appear to be two major uptake mechanisms for CPPs. Endocytosis is an energy-dependent pathway that includes two major categories: phagocytosis involving uptake of large particles, and pinocytosis involving solute uptake [7]. Pinocytosis can be further subdivided into macropinocytosis, and clathrin-dependent, caveolin-dependent and clathrin/caveolin-independent pathways [10]. Endocytosis involves binding to membranes, accumulation in membrane-sunken vesicles, transfer to early and late endosomes, and fusion to become late endosomes/lysosomes [11]–[13]. The progress from binding to transport into early endosomes can be accomplished within 30 min [12], [13]. Direct membrane translocation, also known as direct cell penetration, includes a variety of energy-independent pathways, such as pore formation, inverted micelle formation, carpet-like alternations and membrane thinning [7], [9]. Various physical and pharmacological endocytic inhibitors can be used to identify pathways of CPP-mediated transduction. For instance, low temperature (4°C) treatment arrests all energy-dependent movement across the cell membrane [14]. The endocytic inhibitor cytochalasin D (CytD), an F-actin polymerization disrupter, perturbs endocytic processes that involve clathrin-, caveolae-dependent endocytosis and macropinocytosis [15], [16]. 5-(*N*-ethyl-*N*-isopropyl)-amiloride (EIPA) specifically inhibits macropinocytosis by inhibiting Na^+^/H^+^ exchange proteins [16], [17]. Filipin inhibits lipid raft dependent caveolae endocytosis, while nocodazole inhibits clathrin-dependent endocytosis [15], [16]. The destination of materials internalized by endocytosis is acidic lysosomes, where proteins and other molecules may be degraded by hydrolytic enzymes [18].

Since endocytosis is one of the primary membrane translocation mechanisms of CPPs, escape from endocytic vesicles is essential to preserve biological activity of endocytosed cargoes [7]–[9], [11]. Chloroquine, a lysosomotropic agent, is commonly used to circumvent this problem [19], [20]. Alternatively, peptides with certain sequences can be effective. For instance, the penetration accelerating sequence (Pas) is a synthetic peptide (FFLIPKG) derived from the cleavable sequence (GKPILFF) of cathepsin D enzyme, a lysosomal aspartyl protease [21], [22]. Addition of Pas to octa-arginine (R8), denoted as PasR8, enhances the efficiency of intracellular delivery of bioactive peptides by promoting endosomal escape [21].

Quantum dots (QDs) are colloidal, inorganic nanoparticles with unique chemical and physical properties [23]. They are excellent alternatives to fluorescent proteins due to their high photoluminescent quantum efficiency, photostability, tunability, narrow emission spectral band and prolonged fluorescence lifetime, and they have been extensively used in various cellular imaging applications [23]–[26]. Although QDs can be used to monitor cellular processes, they enter cells very slowly and become entrapped in endosomes [25]–[27]. To overcome these limitations, surface modified QDs by covalent [28]–[30] or noncovalent [16], [31]–[37] linkages with CPPs (referred to as CPP-QD or CPP/QD, respectively) have been introduced.

The aims of this study were to (1) demonstrate Pas nona-arginine (PR9)-mediated cellular internalization of QDs, (2) elucidate the cellular uptake mechanism and subcellular localization of PR9/QD complexes, (3) identify the molecular mechanisms of intracellular trafficking of PR9/QD complexes and (4) identify the physical properties of PR9 and PR9/QD complexes that affect uptake. To achieve these goals, we synthesized PR9 and examined transduction pathways and intracellular shuttling of PR9/QD complexes using flow cytometry and live cell imaging. To identify the cellular uptake mechanisms of PR9 and PR9/QD complexes, pharmacological and physical inhibitors were used to block specific uptake pathways. Transmission electron microscopy (TEM) was used to monitor endocytic progress. Certain physical properties of PR9 and PR9/QD complexes were characterized using zeta-potential analysis and circular dichroism (CD) spectroscopy. The toxicity of PR9 and PR9/QD complexes was assessed using the 1-(4,5-dimethylthiazol-2-yl)-3,5-diphenylformazan (MTT) dye reduction assay. This study provides valuable mechanistic insights into how PR9s promote escape from endocytic vesicles and provides a basis for the design of optimized cargo delivery in cells.

## Materials and Methods

### Cell Culture

Human lung carcinoma A549 cells (American Type Culture Collection, Manassas, VA, USA; CCL-185) were maintained in Roswell Park Memorial Institute (RPMI) 1640 medium (Gibco, Invitrogen, Carlsbad, CA, USA) supplemented with 10% (v/v) bovine serum (Gibco) [38].

### Preparation of Plasmid DNA, Peptides and Nanoparticles

The pEGFP-N1 plasmid contains the enhanced green fluorescent protein (*EGFP*) coding sequence under the control of the cytomegalovirus (CMV) promoter (Clontech, Mountain View, CA, USA) [39], [40]. Three arginine-rich CPPs, nona-arginine (SR9; RRRRRRRRR), histidine-rich nona-arginine (HR9; CHHHHHRRRRRRRRRHHHHHC), and PR9 (FFLIPKGRRRRRRRRR), were chemically synthesized [33]. CdSe/ZnS QDs (denoted as QDs) with a maximal emission peak wavelength at 525 nm (carboxyl-functionalized eFluor 525NC) were purchased from eBioscience (San Diego, CA, USA). Carboxyl-functionalized InP/ZnS QDs (denoted as QInP) with a maximal emission peak wavelength at 525 nm were synthesized [41].

### Protein Transduction and Mechanistic Assay

To analyze the kinetics of protein transduction, A549 cells were seeded at a density of 1×10^5^ per 35-mm petri dish and then incubated overnight in 1 ml of complete growth medium [33]. Six µM CPPs was mixed with 100 nM QDs (i.e., at a molecular ratio of 60) at 37°C for 2 h to form CPP/QD complexes. Cells were washed with 1 ml of phosphate buffered saline (PBS) twice. The cells were then treated with PBS as a control, 100 nM QDs only, or CPP/QD complexes in RPMI 1640 medium supplemented with 1% serum at 37°C for a period of 0–6 h. Cells were washed with PBS five times to remove free CPP/QD complexes before analysis.

To determine the cellular uptake mechanism of PR9/QD complexes, cells were treated with PBS as a negative control, 100 nM QDs alone, or PR9/QD complexes prepared at a molecular ratio of 60 in RPMI 1640 medium supplemented with 1% serum at 37°C for 30 min in the absence or presence of various pharmacological and physical inhibitors [16], [33]. Cells were incubated for 30 min at 4°C to arrest energy-dependent movement across the cell membrane [14], then treated with 100 nM QDs or PR9/QD complexes at 4°C for 30 min. After this treatment, the cells were washed with PBS twice to remove free QDs or PR9/QD complexes, followed by flow cytometric analysis. The influence of modulators on uptake processes was investigated by treating cells with 100 nM QDs or PR9/QD complexes in the absence or presence of 100 µM EIPA, 10 µM CytD, 5 µg/ml filipin, 10 µM nocodazole at 37°C for 30 min. Non-transduced QDs or PR9/QD complexes were removed from cell surface by washing with PBS five times. To assess lysosomal escape, cells were treated with QDs or CPP/QD complexes as described above in the absence or presence of 25 µM chloroquine for 2 h. Free QDs or CPP/QD complexes were excluded by washing with PBS five times before uptake was determined by flow cytometry.

### Flow Cytometric Analysis

Cells were seeded at a density of 1×10^5^ per well in 24-well plates and incubated overnight in 500 µl/well of complete culture medium. Cells in the control and experimental groups treated with QDs or CPP/QD complexes were harvested and counted using a Cytomics FC500 flow cytometer (Beckman Coulter, Fullerton, CA, USA) [33]. To detect green fluorescent proteins (GFP), excitation was set at 488 nm and emission at 515–545 nm with a FL1 filter. Data were analyzed using CXP software (Beckman Coulter). Results are expressed as the percentage of the total cell population that displays fluorescence.

### Subcellular Colocalization Analysis

Endocytic vesicles appear within 30 min, and transport of vesicles toward lysosomes is completed in approximately 2 h [12], [13]. To examine subcellular localization of the delivered PR9/QD complexes, cells were treated with 100 nM green fluorescent QDs alone or CPP/QD complexes in RPMI medium with 1% serum at 37°C for either 30 min or 2 h. Cells were washed with PBS five times to remove free CPP/QD complexes, followed by staining with organelle-specific fluorescent trackers [16], [33]. Treatments with organelle trackers included 16.2 µM Hoechst 33342 (Invitrogen; in blue) at 37°C for 40 min, Texas Red-X phalloidin (Invitrogen; in red) at 37°C for 20 min, 50 nM LysoTracker DND-99 (Invitrogen; in red) at 37°C for 30 min, 50 nM MitoTracker Deep Red FM (Invitrogen; in red) at 37°C for 30 min, and 1 µM ER-Tracker Red (Invitrogen; in red) at 37°C for 30 min to visualize subcellular colocalization with nuclei, actins, lysosomes, mitochondria and endoplasmic reticula (ER), respectively. To investigate intracellular trafficking of PR9/QD complexes, cells were treated with PR9/QD complexes at 37°C from 30 min to 5 h, and then stained with 1,000× diluted rabbit anti-human early endosome antigen 1 protein (EEA1) antibody and goat Alexa Fluor 647-conjugated anti-rabbit antibody fragment (Cell Signaling Technology, Danvers, MA, USA) at 37°C for 12 and 2 h, respectively, to visualize subcellular colocalization with early endosomes.

### Confocal and Fluorescent Microscopy

Fluorescent and bright-field live cell images were recorded using a BD Pathway 435 bioimaging system (BD Biosciences, Franklin Lakes, NJ, USA) equipped with Olympus 20× and 60× oil objectives (Olympus, Tokyo, Japan) [33]. This system includes both confocal and fluorescent microscopy sets. Excitation filters were set at 377/50 nm, 482/35 nm and 543/22 nm for blue (BFP), GFP and red (RFP) fluorescent proteins, respectively. Emission filters were set at 435LP (long-pass), 536/40 nm and 593/40 nm for BFP, GFP and RFP, respectively. Bright-field microscopy was used to assess cell morphology. Intensities of fluorescent images were quantified using BD Pathway software (BD Biosciences).

### Intracellular Trafficking of Different PR9/cargo Complexes

A549 cells were seeded at a density of 1×10^4^ per well in 96-well plates. Cells were treated with PR9/QInP complexes prepared at a molecular ratio of 30 in RPMI 1640 medium supplemented with 1% serum at 37°C from 30 min to 5 h (i.e., all PR9/QD complexes were prepared at a molecular ratio of 60, while all PR9/QInP complexes were formed at a molecular ratio of 30). The solution was then removed, and the cells were washed three times with PBS. The cells were stained with Hoechst 33342 and LysoTracker DND-99 followed by observation using a BD Pathway 435 bioimaging system.

To determine whether PR9 can deliver functional genes, cells were treated with either 3 µg the pEGFP-N1 plasmid DNA alone (control) or a pEGFP-N1 plasmid DNA mixed with PR9 (27 nmole) at a nitrogen (NH_3_^+^)/phosphate (PO_4_^–^) (N/P) ratio of 3 in RPMI 1640 medium supplemented with 1% serum for 30 min to 24 h at 37°C. The solution was then removed, and the cells were washed three times with PBS. The cells were supplemented with 100 µl full growth medium and incubated at 37°C for 48 h. After two days, the cells were stained with Hoechst 33342 and observed using a BD Pathway 435 bioimaging system.

### Transmission Electron Microscopy (TEM)

Morphological examination of PR9/QD- and PR9/QD-transduced cells was performed using a Hitachi H-7500 transmission electron microscope (Hitachi, Tokyo, Japan). PR9/QD complexes were dropped on Formvar/carbon coated copper grids with 300 mesh and dried at room temperature. Cells were treated with PR9/QD complexes, washed five times with PBS to remove free CPP/QD complexes, and then pre-fixed with 2.5% glutaraldehyde in 0.1 M phosphate buffer (pH 7.3) for 1 h. The cells were washed with 0.1 M phosphate buffer twice at a 15-min interval. The cells were post-fixed with 1% osmium for 1 h and washed with 5% sucrose. The cells were stained with or without 2% uranyl acetate, dehydrated in a graded ethanol-acetone series, embedded in Spurr's resin (Electron Microscopy Sciences, Hatfield, PA, USA) [42], and then sliced using a Ultracut-R ultramicrotome (Leica, Wetzlar, Germany). Finally, the cell sections were immobilized on single-well copper grids for TEM analysis.

### Zeta-potential and Particle Size Measurements

QDs (100 nM), PR9 (6 µM) or PR9/QD complexes at a molecular ratio of 60 was dissolved in double deionized water at pH 7 or 5, representing physiological or endosomal conditions, respectively [43], [44]. Each solution was temperature-equilibrated at 25°C for 2 min in a zeta cell. Sizes and zeta-potentials of complexes were measured using a Zetasizer Nano ZS and analyzed using Zetasizer software 6.30 (Malvern Instruments, Worcestershire, UK) [34], [45].

### Circular Dichroism (CD) Spectroscopy

Twenty µM PR9 was dissolved in doubly deionized water with pH 7 or 5 at room temperature. CD spectra of PR9 were analyzed in a cylindrical quartz cuvette with a 1 mm path-length using a Jasco J-715 CD spectrometer (Jasco, Easton, MD, USA) at a scan speed of 50 nm/min [46]. The contents of secondary structures were calculated using CDPro software.

### Cytotoxicity Assay

Cells were seeded at a density of 1×10^4 ^per well in 96-well plates and incubated overnight in 200 µl/well of full growth medium. Cells were treated with PBS as a negative control, treated with 100% dimethyl sulfoxide (DMSO) as a positive control or treated with 25 nM–5 µM QInP in RPMI medium with 1% serum at 37°C for 24 h, washed with PBS, and cultured in full growth medium at 37°C for 48 h. The cells were treated with various concentrations of PR9, individual or combinations of 6 µM PR9, 100 nM QDs, 25 µM chloroquine and 15 µM QInP, as indicated, in RPMI medium with 1% serum at 37°C for 24 h. The cells were washed with PBS and cultured in full growth medium at 37°C for 24 h. Cell viability was measured using the MTT assay [16], [47].

### Statistical Analysis

Data are expressed as mean ± standard deviation (SD). Mean values and SDs were calculated from at least three independent experiments of triplicates per treatment group. Comparisons between the control and treated groups were performed by the Student's *t*-test using levels of statistical significance of *P*<0.05 (*) and 0.01 (**), as indicated.

## Results

### PR9 Displays Cellular Internalization at a Relatively Longer Time

**Figure 1:**
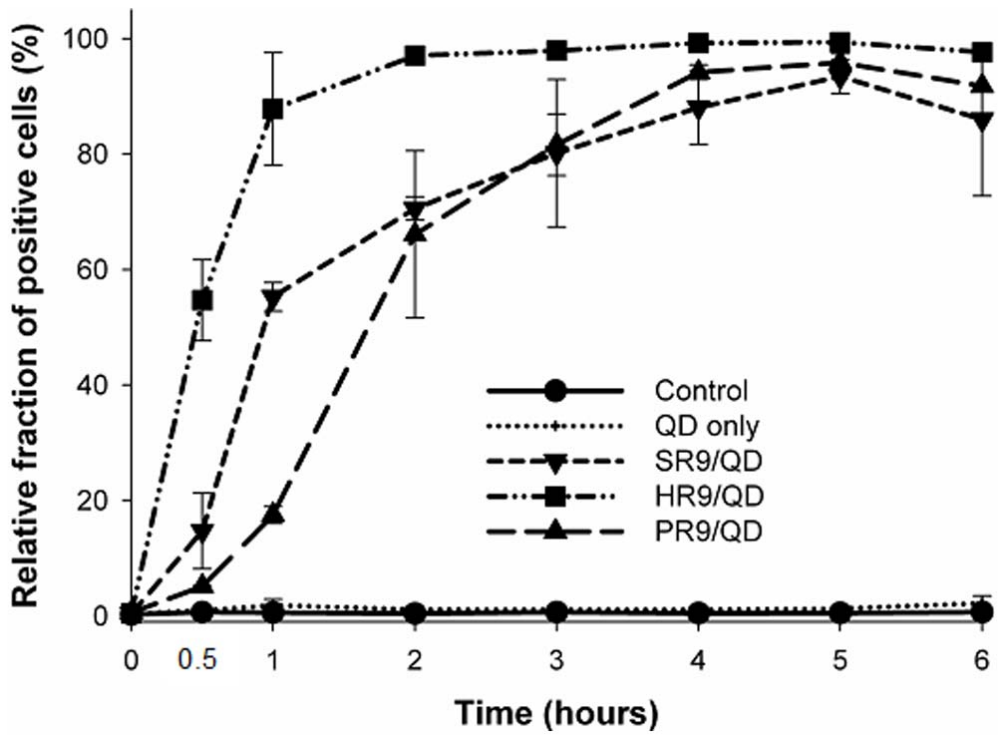
Time course analysis of CPP-mediated cellular internalization of QDs. CPPs, including SR9, HR9 and PR9, were premixed with QDs and then incubated with A549 cells for 0, 0.5, 1, 2, 3, 4, 5 and 6 h. Cellular internalization efficiency was analyzed by flow cytometry. Cells shown green fluorescence were counted as positive signals. Data are presented as mean ± SD from seven independent experiments.

To investigate the efficiency of PR9 mediated transport of QDs into cells, a time course experiment was conducted in human A549 cells. QDs were premixed with or without CPPs SR9, HR9 or PR9 for 2 h, added to cells for up to 6 h, and then analyzed by flow cytometry. At 1 h, the extent of uptake displayed the following order: HR9/QD > SR9/QD > PR9/QD (Figure 1), and uptake of all CPP/QD complexes was essentially complete by 4 h. The differences in uptake kinetics may reflect the involvement of different uptake mechanisms.

### Endocytosis is the Main Pathway for Intracellular Delivery of PR9/QD Complexes

**Figure 2:**
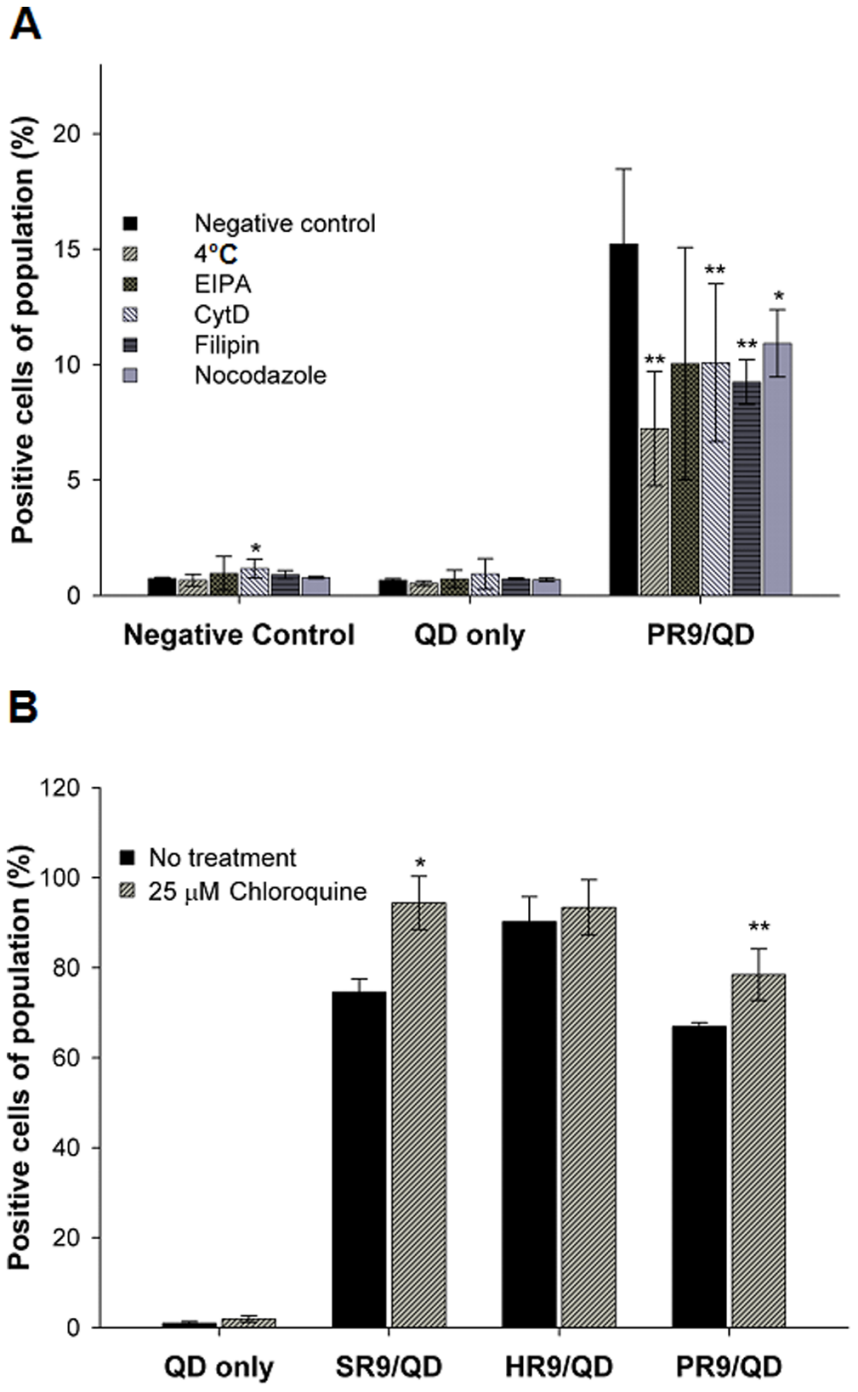
Mechanism of cellular internalization of PR9/QD complexes. (A) Cellular uptake of PR9/QD complexes without or with inhibitors. Cells were treated with PBS as a negative control, QDs alone or PR9/QD complexes in the absence or presence of endocytic inhibitors. Low temperature (4°C), 5-(*N*-ethyl-*N*-isopropyl)-amiloride (EIPA), cytochalasin D (CytD), filipin and nocodazole inhibit different endocytic pathways. Flow cytometry was used to quantitate transduction efficiency. (B) Effect of the lysosomotropic agent chloroquine on cellular uptake of CPP/QD complexes. Significant differences at *P*<0.05 (*) and *P*<0.01 (**) are indicated. Data are presented as mean ± SD from seven (A) and eleven (B) independent experiments.

Various pharmacological and physical inhibitors were used to shed light on the uptake mechanism of PR9/QD complexes. A549 cells were treated with PBS (negative control), QDs alone or PR9/QD complexes in the absence or presence of endocytic inhibitors, followed by flow cytometric analysis. The fraction of cells containing PR9/QD complexes (positive cells) was reduced to 47.5% by incubation at 4°C, to 66.2% by CytD, to 60.7% by filipin, and to 71.7% by nocodazole. In contrast, EIPA treatment (a macropinocytosis inhibitor) did not decrease uptake (Figure 2A). These results indicate that classical energy-dependent endocytosis is the major route for cellular internalization of PR9/QD complexes. To further validate our findings, cells were treated with QDs or CPP/QD complexes with a lysosomotropic agent chloroquine. Chloroquine increased the cellular delivery of SR9/QD and PR9/QD complexes, but not HR9/QD complexes (Figure 2B). This further supports the notion that cellular internalization of PR9/QD complexes involves endocytosis.

### Colocalization of PR9/QD Complexes with Actin Filaments and Lysosomes

**Figure 3:**
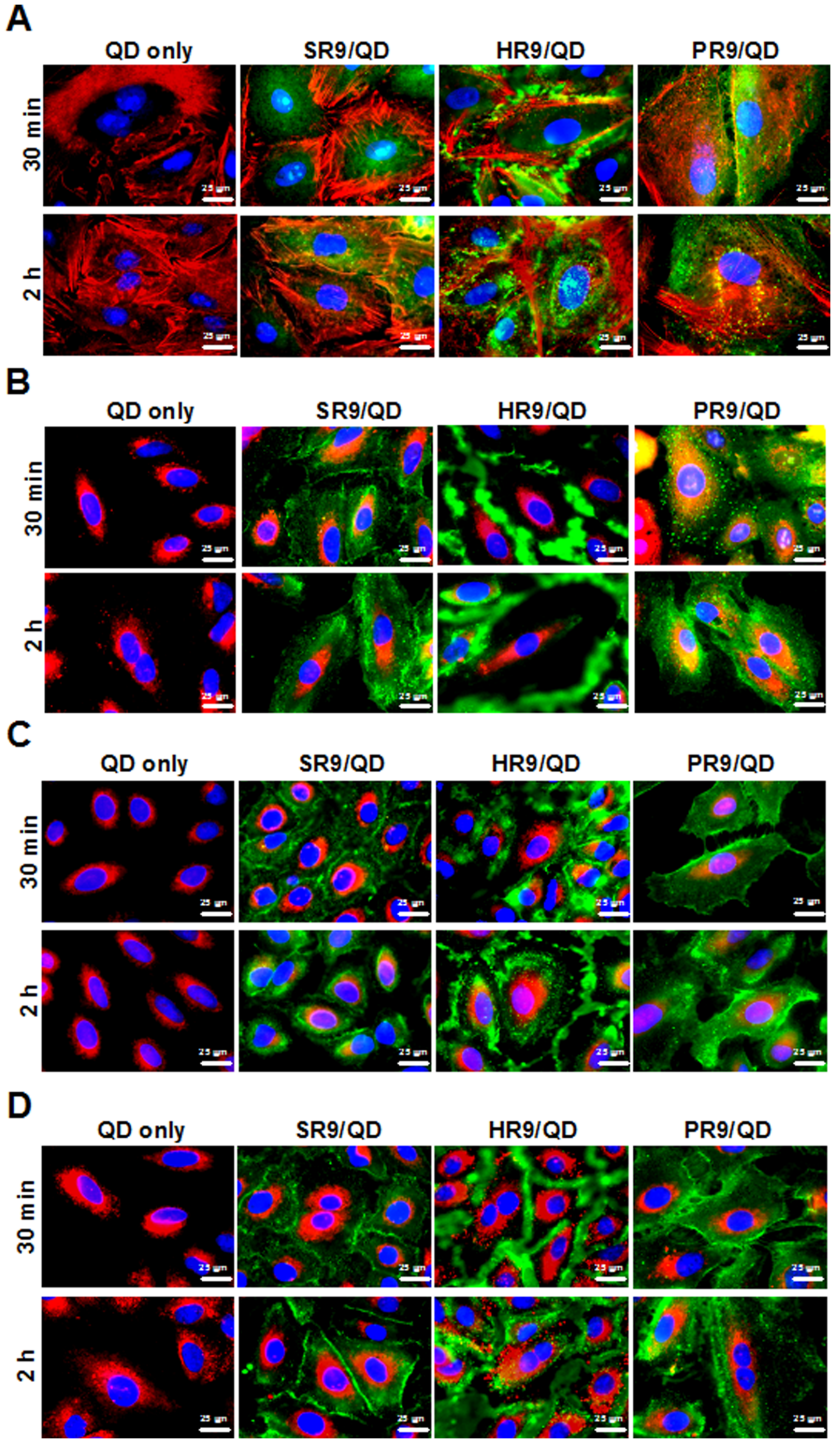
Intracellular colocalization of CPP/QD complexes with organelles. A549 cells were treated with QDs alone, SR9/QD, HR9/QD or PR9/QD complexes for 30 min or 2 h and then stained with Texas Red-X phalloidin for actin filaments (A), LysoTracker DND-99 for lysosomes (B), MitoTracker Deep Red FM for mitochondria (C) or ER-Tracker Red for endoplasmic reticula (ER) (D). Nuclei were stained with Hoechst 33342. Overlap of QDs and organelle trackers are yellow in merged GFP and RFP images. Overlaps of QDs and nuclei are cyan in merged GFP and BFP images. All fluorescent images (A–D) are shown at a magnification of 600×.

To determine the subcellular localization of CPP/QD complexes, cells were treated with QDs or CPP/QD complexes and then stained with organelle-specific fluorescent markers, including Hoechst 33342, Texas Red-X phalloidin, LysoTracker DND-99, MitoTracker Deep Red FM and ER-Tracker Red to visualize nuclei, actins, lysosomes, mitochondria and ER, respectively. Merged images revealed that some PR9/QD complexes colocalized with actins (Figure 3A) and lysosomes (Figure 3B) at 30 min and 2 h. SR9/QD complexes colocalized with actins at 2 h (Figure 3A) and with lysosomes (Figure 3B) at 30 min and 2 h. HR9/QD complexes did not colocalize with any organelles examined (Figure 3A–D). Furthermore, CPP/QD complexes were not associated with mitochondria (Figure 3C), ER (Figure 3D) or nuclei (Figure 3A–D) at any time. These results indicate that cellular internalization of PR9/QD complexes involves classical endocytosis, in agreement with the results presented in Figure 2.

### Intracellular Trafficking of Different PR9/cargo Complexes

The data indicate that PR9/QD complexes are endocytosed. We investigated intracellular trafficking and the fate of the complexes over a period of five hours using signal colocalization of fluorescent CPP/QD complexes with organelle-specific markers.

**Figure 4:**
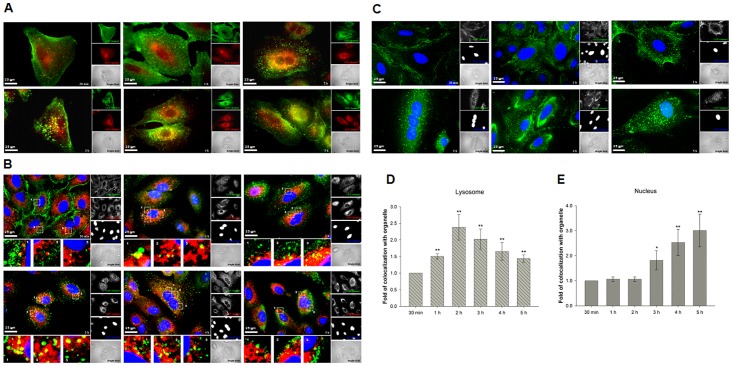
Intracellular trafficking of PR9/QD complexes. (A) Trafficking of PR9/QD complexes toward early endosomes. Cells were treated with PR9/QD complexes for 30 min to 5 h and stained with anti-human early endosome antigen 1 protein (EEA1) antibody. Overlaps of green fluorescent QDs and red fluorescent early endosomes are yellow in merged GFP and RFP images. (B) Trafficking of PR9/QD complexes toward lysosomes. Cells were treated with PR9/QD complexes for 30 min to 5 h and stained with LysoTracker DND-99 and Hoechst 33342. (C) Trafficking of PR9/QD complexes toward the nucleus. A549 cells were treated with PR9/QD complexes for 30 min to 5 h and stained with Hoechst 33342. (D) Time course of colocalization of PR9/QD complexes with lysosomes. (E) Time course of colocalization of PR9/QD complexes with nuclei. Significant differences at *P*<0.05 (*) and *P*<0.01 (**) are indicated. Data are presented as mean ± SD from seven independent experiments. Cell morphology is shown as bright-field images. All fluorescent (A) and confocal images (B and C) are shown at a magnification of 600×.

Cells were treated with PR9/QD complexes for 30 min, 1, 2, 3, 4, and 5 h, followed by staining with anti-human EEA1 antibody, LysoTracker DND-99 and Hoechst 33342. CPP/QD complexes and early endosomes showed limited colocalization at 30 min, and colocalization gradually increased from 1 to 5 h (Figure 4A). Figure 4B indicates that PR9/QD complexes enter lysosomes (see insets 1, 2, 3 obtained at 30 min), and most complexes are trapped in lysosomes at 2–3 h (see insets 1, 2, 3 at 2–3 h). Partially overlapping images between PR9/QD complexes and lysosomes were obtained following longer treatments (insets 1–3 obtained at 4 and 5 h of Figure 4B), suggesting an escape of PR9/QD complexes from the acidic vesicles. High green fluorescent intensity was noticed at the periphery of the nucleus following the longer incubation, indicating an accumulation of PR9/QD complexes in this region (GFP channel at 5 h of Figure 4B and 4 h of Figure 4C). Colocalization intensity of PR9/QD complexes and lysosomes was maximal following a 2 h incubation, and then started to decrease (Figure 4B and D), indicating lysosomal escape.

Figure 4C and E show trafficking of PR9/QD complexes toward nucleus. The complexes spread out near plasma membrane at an early stage and later condense near the nucleus. Localization of PR9/QD complexes in the nucleus was observed from 3 to 5 h (Figure 4B, C and E). Together, these data indicate that after being endocytosed, CPP/QD complexes move sequentially from endosome to lysosome to cytoplasm to nucleus.

**Figure 5:**
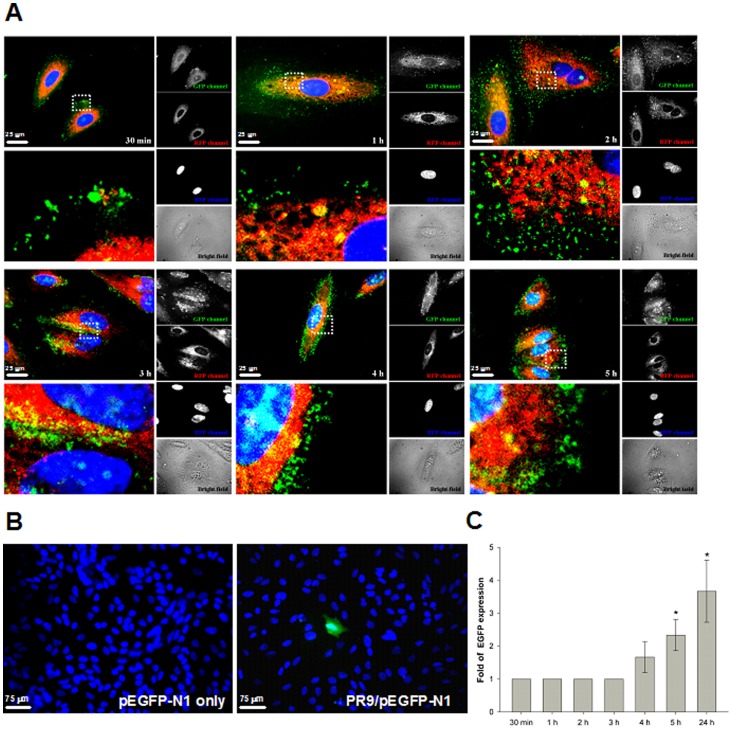
Different cargoes delivered by PR9 follow similar trafficking route in cells. (A) Intracellular trafficking of PR9/QInP complexes. A549 cells were treated with PR9/QInP complexes for 30 min to 5 h and stained with LysoTracker DND-99 and Hoechst 33342. All confocal images are shown at a magnification of 600×. (B) Functional assay of the PR9-delivered plasmid DNA in cells. Cells were treated with pEGFP-N1 plasmid DNA alone or PR9/DNA complexes for 24 h followed by the stain with Hoechst 33342. Green fluorescence revealed cells expressing EGFP (only one of many EGFP-expressing cells is shown here). Blue fluorescence indicates nuclei. Images are shown at a magnification of 200×. (C) Time course of gene expression for PR9/DNA complexes. Significant differences at *P*<0.05 (*) are indicated. Data are presented as mean ± SD from three independent experiments.

To study intracellular trafficking of different PR9/cargo complexes, A549 cells were treated with PR9/QInP complexes for 30 min to 5 h, followed by staining with LysoTracker DND-99 and Hoechst 33342. Trafficking routes of PR9/QInP complexes were recorded at different times. Merged confocal images reveal a colocalization of PR9/QInP complexes with lysosomes and nucleus (Figure 5A). Most PR9/QInP complexes were trapped in lysosomes at 2 h, but exited the lysosomes and translocated toward the nucleus at 2–5 h (Figure 5A). To demonstrate delivery of PR9/cargo complexes to the nucleus, a functional gene assay was carried out. Cells were treated with pDNA alone or PR9/DNA complexes for 30 min, 1–5 and 24 h, followed by staining with Hoechst 33342. After a 24 h incubation, no fluorescence was detected in the cells treated with pDNA only (Figure 5B). In contrast, green fluorescence was apparent in cells treated with PR9/DNA complexes at later times (Figure 5B and C), indicating that plasmid DNA delivered by PR9s can be expressed. These results demonstrate that different cargoes delivered by PR9 follow a similar trafficking pattern in cells. The functional gene assay confirmed the colocalization of cargoes with the nucleus at a later stage.

### TEM

**Figure 6:**
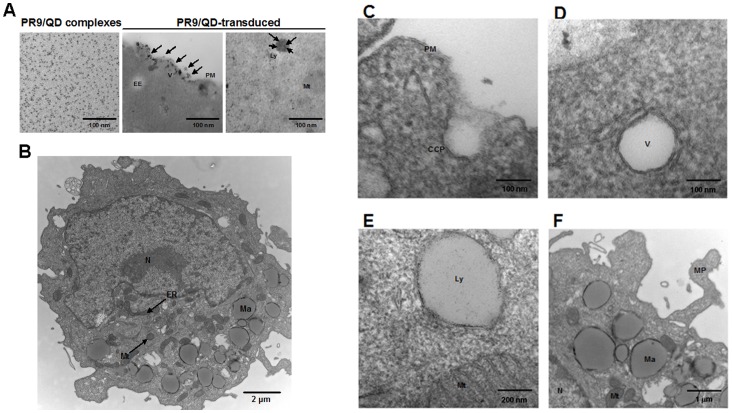
Transmission electron microscopy (TEM) images of PR9/QD complexes. (A) Images of PR9/QD complexes and PR9/QD-transduced cells. PR9 peptide was mixed with QDs at a molecular ratio of 60 (left). Cells were treated with PR9/QD complexes; arrows indicate the location of PR9/QD complexes (middle and right). (B–F) Images of PR9/QD-transduced cells. CCP = clathrin-coated pit, EE = early endosome, ER = endoplasmic reticulum, Ly = lysosome, Ma = macropinosome, MP = membrane protrusion, Mt = mitochondrium, N = nucleus, PM = plasma membrane, V = vesicle.

The morphology of PR9/QD complexes and PR9/QD-transduced cells was observed using a Hitachi TEM. PR9/QD complexes were spherical with an average diameter of 2.0±0.1 nm (Figure 6A left). Electron-dense cores of PR9/QD complexes were observed associated with the membranes of PR9/QD-transduced cells following a 30-min incubation (Figure 6A middle). PR9/QD complexes were observed in a lysosome after a 2-h incubation (Figure 6A right). The endocytic progress of PR9/QD complexes was indicated by the appearance of labeled macropinosomes and lysosomes in PR9/QD-transduced cells (Figure 6B). PR9/QD complexes on the membrane were observed enclosed in a clathrin-coated pit (Figure 6C), and later enclosed within a vesicle (early endosome) (Figure 6D). PR9/QD complexes-containing macropinosomes, which ultimately fuse with lysosomes (Figure 6E), were noted in lamellipodia-like membrane protrusions (Figure 6F). These results indicate that PR9 transports QDs into cells by an endocytic pathway.

### Zeta-potential, Particle Size Measurements and CD Spectroscopy

**Figure 7:**
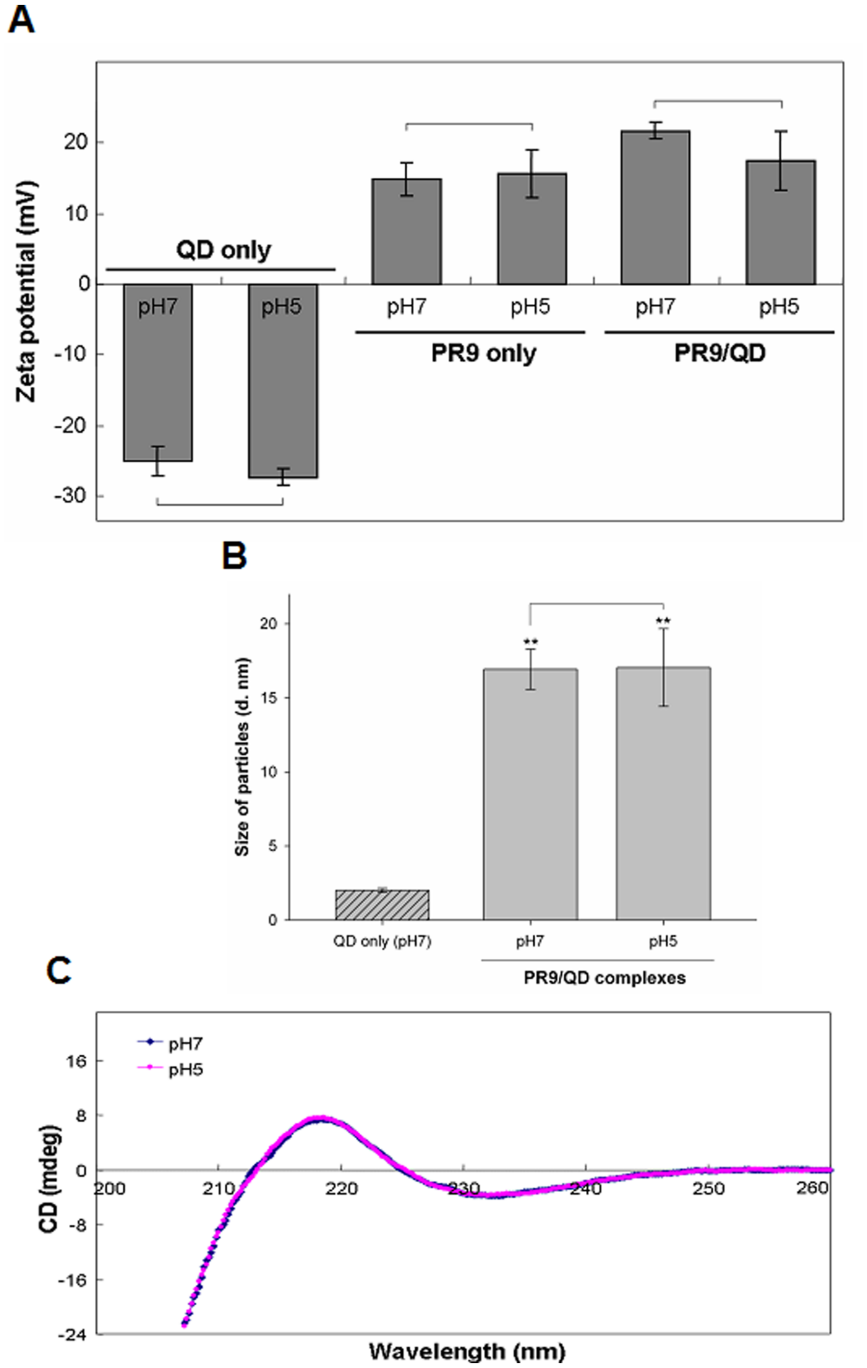
Zeta-potential and particle size of PR9 and PR9/QD complexes and the secondary structure of PR9. (A) Zeta-potentials of PR9 and PR9/QD complexes. PR9 or PR9/QD complexes prepared at a molecular ratio of 60 were dissolved in doubly deionized water at pH 7 or 5. Each solution was equilibrated at 25°C for 120 sec in a zeta cell and then analyzed using a Zetasizer Nano ZS. (B) Particle size of QD or PR9/QD complexes. PR9/QD complexes were dissolved in doubly deionized water with pH 7 or 5 and then analyzed using a Zetasizer. Significant differences between PR9/QD complexes and QDs at *P*<0.01 (**) are indicated. Data are presented as mean ± SD from seven independent experiments. (C) Secondary structure of PR9. All CD spectra were recorded in millidegree (mdeg).

To characterize the physical properties of PR9 and PR9/QD complexes, zeta-potential, particle size and secondary structure of PR9 were determined. PR9 and PR9/QD complexes were analyzed in aqueous solutions at pH 7 (cytosolic condition) and pH 5 (lysosomal condition). Zeta-potentials of QDs were electronegative at pH 7 (−25.1±2.2 mV) and pH 5 (−27.3±1.2 mV) (Figure 7A); zeta-potentials of arginine-rich PR9 were 14.9±2.4 mV at pH 7, and 15.6±3.4 mV at pH 5. PR9/QD complexes were even more positive: 21.7±1.1 mV at pH 7 and 17.4±4.2 mV at pH 5, conditions favoring protein transduction. On the other hand, PR9/QD complexes were of similar sizes: 16.9±1.4 and 17.1±2.6 nm at pH 7 and 5, respectively, while PR9/QD complexes were larger than QDs (Figure 7B). These results agree with our recent report that the electrostatic interactions of CPP/cargo complexes can be a predictor of transduction efficiency within the charge range tested [48], emphasizing the significance of zeta-potential for the transduction of PR9/QD complexes.

To determine whether conformational changes of PR9 in acidic vesicles could be a factor in lysosomal escape, the secondary structure of PR9 was analyzed using CD spectroscopy. PR9 conformations were very similar at pH 7 and 5 (Figure 7C). Collectively, these results indicate that zeta-potentials of CPP/QD complexes are a key factor of transduction efficiency [48], reflecting the importance of electrostatic interactions of PR9/QD complexes with plasma membranes and endomembrane system.

### Cytotoxicity

**Figure 8:**
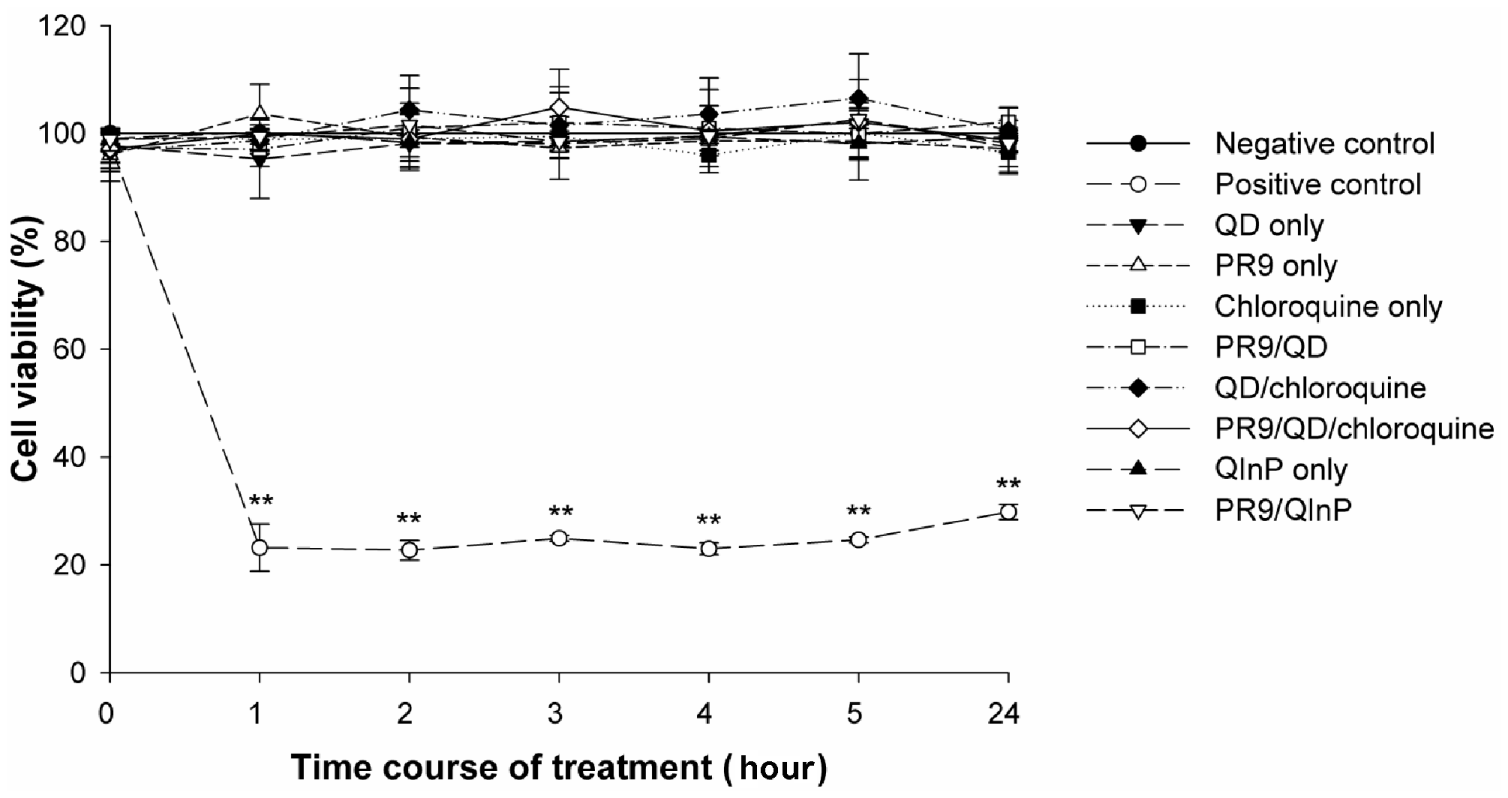
Cytotoxicity of materials used for cargo delivery by PR9 as determined using the MTT assay. Cells were treated with QD, PR9, chloroquine, PR9/QD, QD/chloroquine, PR9/QD/chloroquine, QInP alone and PR9/QInP complexes for 24 h. Significant differences at *P*<0.05 (*) and *P*<0.01 (**) are indicated. Data are presented as mean ± SD from three independent experiments.

The MTT assay was used to determine the effect of PR9-mediated cargo delivery on cell viability. Cells were treated with either individual or a combination of PR9s, QDs, chloroquine and QInP, as indicated. None of the materials used in this study caused cytotoxicity (Figure 8), indicating that arginine-rich PR9 would be a safe vehicle to carry cargo into cells.

## Discussion

In this study, we demonstrate that classical energy-dependent endocytosis is the major route for cellular internalization of PR9/QD complexes, and that chloroquine exerts a lysosomotropic effect on PR9/QD complexes, allowing them to escape from endocytic vesicles into the cytoplasm. PR9/QD complexes colocalize with actins, lysosomes, early endosomes and nucleus. TEM analysis revealed the endocytic trafficking of PR9/QD complexes in cells. A reporter gene assay confirmed that plasmid DNA delivered by PR9s can be actively expressed by cells [49]. Zeta-potentials of CPP/cargo complexes correlated with transduction efficiency [48], emphasizing the importance of electrostatic interactions of PR9/QD complexes with plasma membranes. Cell viability assay confirmed that none of the components of the PR9/QD complexes are cytotoxic.

CPP transduction of QDs into stem cells with high transduction efficiency and low cytotoxicity has been demonstrated [37], [50], [51]. Moreover, we have shown that PR9 and PR9/cargo complexes are relatively nontoxic in A549 cells by SRB [33] and MTT [52], [53] assays. Our present results with PR9/QD complexes in human cells are consistent with these earlier results. It was reported that more than 80% of adipose tissue-derived stem cells could be labeled by R8/QD complexes prepared at a ratio of 10,000 within 1 h, and that the consequent fluorescent staining was maintained at least for 2 weeks [37]. No cytotoxicity was observed in cells transduced with less than 16 nM of QDs. In addition, the transduced cells could differentiate into adipogenic and osteogenic cells, indicating that the transduced cells maintained their stem cell potency [37].

Research on CPPs has focused on improving transduction efficiency. The hybrid PasR8 peptide markedly enhanced the translocation efficiency of active peptides by permitting endosomal escape in cells [21]. For instance, Pas conjugated with flock house virus (FHV)-derived arginine-rich peptide was attached to the p53 C-terminal 22-amino-acid peptide (p53C'), a retro-inverso peptide that induces p53-dependent autophagic cell death [54]. In another study, the growth of malignant glioma cells was inhibited by the triplex D-isomer peptides (dPasFHV-p53C'). Recently, the importance of hydrophobic sequences in the Pas segment, especially phenylalanine residues, in promoting cellular uptake of R8 was demonstrated [55]. Attachment of aromatic moieties, such as Pas, to a R8 segment may increase peptide-proteoglycan interactions, thereby stimulating macropinocytosis. PasR8 working in a serum-containing medium was an additional advantage of the Pas segment, since serum-binding often decreases cytosolic internalization of CPPs. The promotion of cellular uptake by Pas addition is prominent when the molecular weight of cargoes is relatively small. Finally, the total hydrophobicity of PasR8 conjugates appears to be crucial for efficient cytosolic translocation [55].

TEM is a valuable tool for the morphological characterization of biological and nonbiological materials at high resolution [56]. Direct information on the intracellular distribution of transduced material comes from TEM, which reveals electron-dense cores of PR9/QD complexes associated with plasma membrane and in the cytoplasm of PR9/QD-transduced cells (Figure 6). While there are multiple types of endocytic pathways [15], the endocytic progress of transport vesicles of the widely studied clathrin-dependent endocytosis of nanoparticles is from early endosomes to multivesicular bodies/late endosomes and finally to lysosomes. TEM images of PR9/QD-transduced cells obtained in the present study were generally in accord with this endocytic progression. PR9/QD complexes were somewhat larger than QDs alone (Figure 7B), suggesting that positively charged PR9s form stable complexes with carboxyl-functionalized QDs by electrostatic interactions [16], [33].

Zeta-potential is a useful measure in nanoparticle applications that indicates the interaction energy on the particle-carrier surface [57], [58]. Zeta-potential depends on nanoparticle size, methods of production and treatment, surface structure and the pH value of the environment [37]. The combined effects of both zeta-potential and particle size on nanoparticles provide insight into the stability of particles in solution [59], [60]. We found that more electropositive zeta values of CPP/cargo complexes correlate well with protein transduction efficiency, presumably due to increased electrostatic interactions of PR9/QD complexes with plasma membranes. In this study, the more electropositive PR9/QD complexes had a higher transduction efficiency than PR9s or QDs.

Qualitative secondary structure assignments of CD spectroscopy were based on the following: minima at both 208 and 222 nm, and maximum at 190 nm for α-helix; minimum at 218 nm and maximum at 195 nm for β-sheet; minimum at 198 nm and no positive peak for random coil [61]. We found that the secondary structural contents of PR9 have very similar conformations in aqueous buffers at pH 7 and pH 5 (Figure 7C). These two patterns (minimum at 198–222 nm and maximum) of PR9 are similar to those of R9, which is mostly unstructured in solution [62]. Binding of poly-L-arginine composed of 293 (PLA239) and 554 (PLA554) arginine-residues to an anionic phospholipid large unilamellar vesicle (LUV) was accompanied by a transition from random coil to α-helix structure; however, a similar structural change was not observed with PLA69 and R8 [63].

Subcellular colocalization analyses revealed that HR9/QD complexes do not colocalize with any organelles tested; these complexes stay in the cytosol most of time (Figure 3). This result is consistent with our earlier demonstration that HR9/QD complexes enter cells by direct membrane translocation [33], [34]. In contrast, endocytosis appears to be the main route for intracellular delivery of PR9/QD and SR9/QD complexes [34]. However, SR9/QD complexes entered cells by multiple pathways [64]. Among them, macropinocytosis, a lipid raft-dependent form of endocytosis, is a prominent route for SR9/QD entry [65]. Actin forms microfilaments, one of key components of the cytoskeleton, participating in many cellular processes, including endocytosis. Macropinocytosis and classical endocytosis, such as clathrin-, caveolae-dependent, and clathrin- and caveolae-independent pathways, involve actin rearrangements. Therefore, the observed colocalization of PR9/QD with actin, lysosomes and early endosomes, indicates that these complexes enter cells through an endocytic pathway.

Numerous factors, including experimental conditions, physicochemical properties of CPPs and their cargoes, cell type, temperature and serum level in the medium can influence the pathway of cellular uptake [7], [11], [66]–[70]. R9, antennapedia peptide and Tat peptide use a combination of three endocytic pathways: macropinocytosis, clathrin-mediated endocytosis and caveolae/lipid-raft-mediated endocytosis [66]. It seems likely that PR9s use the same three endocytic pathways (Figure 2A and 3–6). The chemical properties of the cargo molecules are a contributing factor of dodeca-arginine (R12) peptide-mediated translocation [69]. R12 attached to hydrophobic cargoes stimulate dynamic morphological alternations in plasma membranes, and these structural changes allow R12 to permeate plasma membranes [69].

### Conclusions

PR9/QD complexes comprised of a cell penetrating peptide (nona-arginine; R9) and a peptide penetration accelerating sequence (Pas) that promotes lysosomal escape noncovalently complexed with a quantum dot (QD) probe were evaluated as a cell transduction system. Histological results demonstrate that endocytosis is the main pathway for cellular uptake of PR9/QD complexes. PR9/QD complexes initially colocalize with actins, lysosomes and early endosomes, and later with the nucleus. Plasmid DNA delivered by PR9s was expressed by cells. Zeta-potential analysis revealed the importance of electrostatic interactions of PR9/QD complexes with plasma membranes. PR9/QD complexes were not toxic to the cells. Thus, PR9 may be an efficient and safe delivery vector for biomedical applications.
